# Supplementary material for: Insights into Species Preservation: Cryobanking of Rabbit Somatic and Pluripotent Stem Cells
Source: Int J Mol Sci. 2020 Oct 2;21(19):7285. doi: 10.3390/ijms21197285 (PMC7582889; doi:10.3390/ijms21197285)
Supplement: Supplementary file 1 [file ijms-21-07285-s001.zip › supplementary files ijms-903345-revised/Lucie-TableS3.docx]

**Table S3: rbF infection rates with the GFP-expressing Sendai vector**

**A: rbF infection rates according to animals**

|  | **rbF frozen controls** | | | | **Frozen tissue-derived rbF** | | | | | | | | | | | | **rbF fresh controls** | | | | | |
| --- | --- | --- | --- | --- | --- | --- | --- | --- | --- | --- | --- | --- | --- | --- | --- | --- | --- | --- | --- | --- | --- | --- |
|  | **Rabbit 1** | | **Rabbit 2** | | **Rabbit 4** | | **Rabbit 5** | | **Rabbit 6** | | **Rabbit 7** | | **Rabbit 8** | | **Rabbit 9** | | **Rabbit 10** | | **Rabbit 11** | | **Rabbit 12** | |
|  | **N°S*** | **%I°** | **N°S** | **%I** | **N°S** | **%I** | **N°S** | **%I** | **N°S** | **%I** | **N°S** | **%I** | **N°S** | **%I** | **N°S** | **%I** | **N°S** | **%I** | **N°S** | **%I** | **N°S** | **%I** |
|  | 71 | 18.7 | 73 | 29.9 | 7 | 50.7 | 15 | 44.0 | 23 | 93.2 | 31 | 68.5 | 55 | 94.5 | 63 | 61.8 | 1 | 45.4 | 3 | 32.6 | 5 | 18.3 |
|  | 72 | 10.7 | 74 | 22.4 | 8 | 43.9 | 16 | 65.0 | 24 | 93.7 | 32 | 69.5 | 56 | 92.7 | 65 | 92.0 | 2 | 18.3 | 4 | 7.7 | 6 | 18.3 |
|  |  |  |  |  | 9 | 74.6 | 17 | 77.8 | 25 | 94.2 | 33 | 66.6 | 57 | 94.4 | 66 | 93.2 |  |  |  |  |  |  |
|  |  |  |  |  | 11 | 61.7 | 18 | 80.3 | 26 | 94.0 | 35 | 71.1 | 58 | 30.4 | 68 | 86.1 |  |  |  |  |  |  |
|  |  |  |  |  | 12 | 52.3 | 19 | 77.0 | 27 | 66.6 | 36 | 69.6 | 60 | 76.5 | 69 | 88.5 |  |  |  |  |  |  |
|  |  |  |  |  | 13 | 44.0 | 21 | 77.2 | 28 | 73.3 | 37 | 66.5 | 61 | 22.9 |  |  |  |  |  |  |  |  |
|  |  |  |  |  | 14 | 49.0 | 22 | 74.3 | 29 | 69.8 | 38 | 73.9 | 62 | 46.8 |  |  |  |  |  |  |  |  |
|  |  |  |  |  |  |  |  |  | 30 | 49.5 | 48 | 19.9 |  |  |  |  |  |  |  |  |  |  |
|  |  |  |  |  |  |  |  |  | 43 | 50.3 | 50 | 56.6 |  |  |  |  |  |  |  |  |  |  |
|  |  |  |  |  |  |  |  |  | 45 | 29.1 | 53 | 61.7 |  |  |  |  |  |  |  |  |  |  |
| **Mean** |  | **14.7** |  | **26.2** |  | **53.7** |  | **70.8** |  | **71.4** |  | **62.5** |  | **65.5** |  | **84.4** |  | **31.9** |  | **20.2** |  | **18.3** |
| SD |  | 5.7 |  | 5.3 |  | 10.2 |  | 12.8 |  | 22.9 |  | 16.7 |  | 31.5 |  | 12.9 |  | 19.2 |  | 17.6 |  | 0.0 |

**B: rbF infection rates according to tissues**

| **Sample types** | |  |  |  |  |  |  |  |  |  |  |  |  |  |  |  |  |  |  |  |  |  |  |  |  | **Mean** | SD |
| --- | --- | --- | --- | --- | --- | --- | --- | --- | --- | --- | --- | --- | --- | --- | --- | --- | --- | --- | --- | --- | --- | --- | --- | --- | --- | --- | --- |
| **rbF**  **controls** | **S^1^** | **N°S*** | 1 | 3 | 5 | 71 | 72 |  |  |  |  |  |  |  |  |  |  |  |  |  |  |  |  |  |  |  |  |
|  |  | **%I°** | 45.4 | 32.6 | 18.3 | 18.7 | 29.9 |  |  |  |  |  |  |  |  |  |  |  |  |  |  |  |  |  |  | **29.0** | 11.2 |
|  | **C^2^** | **N°S** | 2 | 4 | 6 | 72 | 74 |  |  |  |  |  |  |  |  |  |  |  |  |  |  |  |  |  |  |  |  |
|  |  | **%I** | 18.3 | 7.7 | 18.3 | 10.7 | 22.4 |  |  |  |  |  |  |  |  |  |  |  |  |  |  |  |  |  |  | **15.5** | 6.1 |
| **Frozen tissue-derived rbF** | **S** | **N°S** | 7 | 8 | 9 | 15 | 16 | 17 | 18 | 23 | 24 | 25 | 26 | 31 | 32 | 33 | 48 | 50 | 55 | 56 | 57 | 58 | 63 | 65 | 66 |  |  |
|  |  | **%I** | 50.7 | 43.9 | 74.6 | 44.0 | 65.0 | 77.8 | 80.3 | 93.2 | 93.7 | 94.2 | 94.0 | 68.5 | 69.5 | 66.6 | 19.9 | 56.6 | 94.5 | 92.7 | 94.4 | 30.4 | 61.8 | 92.0 | 93.2 | **72.1** | 23.0 |
|  | **C** | **N°S** | 11 | 12 | 13 | 14 | 19 | 21 | 22 | 27 | 28 | 29 | 30 | 35 | 36 | 37 | 38 | 43 | 45 | 53 | 60 | 61 | 62 | 68 | 69 |  |  |
|  |  | **%I** | 61.7 | 52.3 | 44.0 | 49.0 | 77.0 | 77.2 | 74.3 | 66.6 | 73.3 | 69.8 | 49.5 | 71.1 | 69.6 | 66.5 | 73.9 | 50.3 | 29.1 | 61.7 | 76.5 | 22.9 | 46.8 | 86.1 | 88.5 | **62.5** | 17.0 |

*Number of sample (rbF line)

°Percentage of infection (percentage of GFP-positive cells detected by flow cytometry)

^1^S = Skin; ^2^C = Cartilage
