# Supplementary material for: Insights into Species Preservation: Cryobanking of Rabbit Somatic and Pluripotent Stem Cells
Source: Int J Mol Sci. 2020 Oct 2;21(19):7285. doi: 10.3390/ijms21197285 (PMC7582889; doi:10.3390/ijms21197285)
Supplement: Supplementary file 1 [file ijms-21-07285-s001.zip › supplementary files ijms-903345-revised/Lucie-TableS2.docx]

**Table S2: Composition of freezing media**

| **Tissues or**  **cell lines** | **Media for unicellular suspension*** | **Basal media for freezing** | **DMSO concentration in freezing media (v/v, %)** | **Final DMSO concentration**  **(v/v, %)** |
| --- | --- | --- | --- | --- |
| Skin or cartilage pieces | / | FBS | 4 or 10 | 4 or 10 |
|  |  | CRYO3 | 4 or 10 | 4 or 10 |
| rbFs | rbF medium | FBS | 20 | 10 |
| mESCs | mESC medium | FBS | 0, 5, 6, 8, 10 or 20 | 0, 2.5, 3, 4, 5 or 10 |
|  | CRYO3 | CRYO3 | 0, 5, 6, 8, 10 or 20 | 0, 2.5, 3, 4, 5 or 10 |
|  | CryoStor® CS10 | / | 10 | 10 |
| rbESCs | rbESC medium | FBS | 0, 5, 6, 8, 10 or 20 | 0, 2.5, 3, 4, 5 or 10 |
|  | CRYO3 | CRYO3 | 0, 5, 6, 8, 10 or 20 | 0, 2.5, 3, 4, 5 or 10 |
|  | CryoStor® CS10 | / | 10 | 10 |
| rbiPSCs | rbiPSC medium | FBS | 0, 5, 6, 8, 10 or 20 | 0, 2.5, 3, 4, 5 or 10 |
|  | CRYO3 | CRYO3 | 0, 5, 6, 8, 10 or 20 | 0, 2.5, 3, 4, 5 or 10 |
|  | CryoStor® CS10 | / | 10 | 10 |

*Dissociated cells are resuspended in culture medium or CRYO3 without DMSO (50% volume final) before adding 50% volume of corresponding freezing medium, excepted for ready-to-used CryoStor® CS10, cells are then directly resuspended in this ready-to-used freezing medium.
