# Supplementary material for: Insights into Species Preservation: Cryobanking of Rabbit Somatic and Pluripotent Stem Cells
Source: Int J Mol Sci. 2020 Oct 2;21(19):7285. doi: 10.3390/ijms21197285 (PMC7582889; doi:10.3390/ijms21197285)
Supplement: Supplementary file 1 [file ijms-21-07285-s001.zip › supplementary files ijms-903345-revised/Lucie-TableS4.docx]

**Table S4: Primers used for RT-qPCR analyses**

| **Gene** | **Species** | **Direction** | **Primers** |
| --- | --- | --- | --- |
| *OCT4* | Rabbit | Forward | CCTGCTCTGGGCTCCCCCAT |
|  |  | Reverse | TGACCTCTGCCTCCACCCCG |
| *NANOG* | Rabbit | Forward | CACTGATGCCCGTGGTGCCC |
|  |  | Reverse | AGCGGAGAGGCGGTGTCTGT |
| *ESRRB* | Rabbit | Forward | CGTGGAGGCCGCCAGAAGTA |
|  |  | Reverse | TCTGGCTCGGCCACCAAGAG |
| *REX1 (ZFP42)* | Rabbit | Forward | AGCCCAGCAGGCAGAAATGGAA |
|  |  | Reverse | TGGTCAGTCTCACAGGGCACAT |
| *CDH1* | Rabbit | Forward | TGCACAGGCCGGAAACCAGT |
|  |  | Reverse | ACGGCCTTCAGCGTGACCTT |
| *CDH2* | Rabbit | Forward | CCGTGGCAGCTGGACTGGAT |
|  |  | Reverse | GATGACGGCCGTGGCTGTGT |
| *TBP* | Rabbit | Forward | CTTGGCTCCTGTGCACACCATT |
|  |  | Reverse | ATCCCAAGCGGTTTGCTGCTGT |
| *Oct4* | Mouse | Forward | ATGCAAATCGGAGACCCTGGTGC |
|  |  | Reverse | AGCCCAAGCTGATTGGCGATGTG |
| *Nanog* | Mouse | Forward | AAGCCATGCGCATTTTAGCACCC |
|  |  | Reverse | AAGGAACCTGGCTTTGCCCTGAC |
| *Esrrb* | Mouse | Forward | CTCGCCAACTCAGATTCGAT |
|  |  | Reverse | AGAAGTGTTGCACGGCTTTG |
| *Rex1 (Zfp42)* | Mouse | Forward | TGTGTGCAGAGTGTGGCAAAGC |
|  |  | Reverse | TGGGTGCGCAAGTTGAAATCCAG |
| *Actb* | Mouse | Forward | TTCTTTGCAGCTCCTTCGTTGCC |
|  |  | Reverse | TTTGCACATGCCGGAGCCGTTG |
