# Supplementary material for: Insights into Species Preservation: Cryobanking of Rabbit Somatic and Pluripotent Stem Cells
Source: Int J Mol Sci. 2020 Oct 2;21(19):7285. doi: 10.3390/ijms21197285 (PMC7582889; doi:10.3390/ijms21197285)
Supplement: Supplementary file 1 [file ijms-21-07285-s001.zip › supplementary files ijms-903345-revised/Lucie-TableS1.docx]

**Table S1: Effect of tissue treatments* and freezing conditions** on rbF derivation°**

**Short fibroblasts**

**Elongated fibroblasts**

**Mix of morphologies**

**Epithelial-type cells**

| **Skin** | | | | | **Cartilage** | | | | |
| --- | --- | --- | --- | --- | --- | --- | --- | --- | --- |
| **rbF morphologies** | **FBS** | | **CRYO3** | | **rbF morphologies** | **FBS** | | **CRYO3** | |
|  | **4%** | **10%** | **4%** | **10%** |  | **4%** | **10%** | **4%** | **10%** |
| 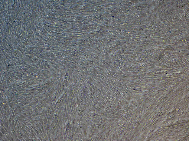 40 | 40 (23d) | 7 (22d)  23 (20d)  55 (18d)  63 (20d) |  | 9 (20d)  25 (20d)  33 (22d)  41 (23d)  49 (22d)  57 (20d)  65 (20d) | 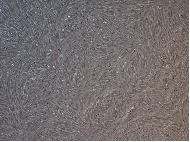 44 | 44 (19d) |  | 38 (22d) |  |
| 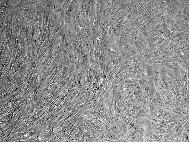 16 | 16 (25d)  32 (22d)  48 (21d) |  | 18 (22d)  26 (28d)  50 (22d)  66 (20d) | 17 (20d) | 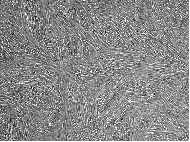 19 | 36 (18d) | 11 (18d)  19 (18d)  35 (18d) | 22 (22d) | 13 (20d)  21 (21d)  29 (22d)  37 (18d)  45 (20d) |
| 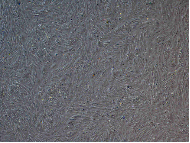 58 | 56 (20d) | 15 (18d) | 58 (20d) |  | 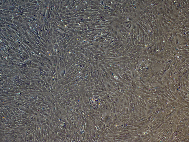 46 | 20 (18d)  60 (17d) | 43 (19d) | 14 (25d)  46 (21d)  62 (20d) | 53 (19d)  61 (17d) |
| 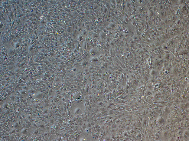 8 | 8 (22d)  24 (20d) | 31 (18d) |  |  | 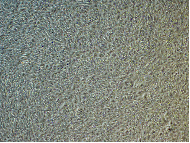 28 | 12 (25d)  28 (21d)  68 (20d) | 27 (18d) | 30 (18d) | 69 (20d) |

* Direct treatment of 3 mm^2^ pieces / Direct treatment of 1 cm^2^ pieces / Treatment after 48h at 4°C of 3 mm^2^ pieces

** Freezing media: FBS or CRYO3 + 4% or 10% DMSO

° Times (d = days) of rbF derivation (2 passages before freezing) are noticed in brackets after the line numbers, and are equal on average to 14 and 16 days for fresh cartilage and skin samples, respectively.
